# Time course of lung ultrasound findings in patients with COVID-19 pneumonia and cardiac dysfunction

**DOI:** 10.1186/s13089-022-00278-2

**Published:** 2022-07-07

**Authors:** Joao Leote, Tiago Judas, Ana Luísa Broa, Miguel Lopes, Francisca Abecasis, Inês Pintassilgo, Afonso Gonçalves, Filipe Gonzalez

**Affiliations:** 1 Critical Care Department, Hospital Garcia de Orta E.P.E, Av. Torrado da Silva, 2805-267 Almada, Portugal; 2 Internal Medicine Department, Hospital Garcia de Orta E.P.E, Av. Torrado da Silva, 2805-267 Almada, Portugal; 3 Pulmonology Department, Hospital Garcia de Orta E.P.E, Av. Torrado da Silva, 2805-267 Almada, Portugal; 4 Radiology Department, Hospital Garcia de Orta E.P.E, Av. Torrado da Silva, 2805-267 Almada, Portugal

**Keywords:** LUS, Ultrasound, COVID-19, Pneumonia, Cardiac dysfunction

## Abstract

**Background:**

Lung ultrasound (LUS) is a valuable tool to predict and monitor the COVID-19 pneumonia course. However, the influence of cardiac dysfunction (CD) on LUS findings remains to be studied. Our objective was to determine the effect of CD on LUS in hospitalized patients with COVID-19 pneumonia.

**Material and methods:**

Fifty-one patients with COVID-19 pneumonia participated in the study. Focused echocardiography (FoCUS) was carried out on day 1 to separate patients into two groups depending on whether they had FoCUS signs of CD (CD+ vs CD−). LUS scores, based on the thickness of the pleural line, the B-line characteristics, and the presence or not of consolidations, were obtained three times along the patient’s admission (D1, D5, D10) and compared between CD+ and CD− patients. A correlation analysis was carried out between LUS scores and the ratio of the arterial partial pressure of oxygen to the fraction of the inspired oxygen (P/F ratio).

**Results:**

Twenty-two patients were CD+ and 29 patients were CD−. Among the CD+ patients, 19 were admitted to the intensive care unit (ICU), seven received invasive mechanical ventilation (IMV), and one did not survive. Among the CD− patients, 11 were admitted to the ICU, one received IMV and seven did not survive. CD+ patients showed a significantly lower P/F ratio than CD− patients. However, LUS scores showed no between-group differences, except for fewer subpleural consolidations in the upper quadrants of CD+ than on CD− patients.

**Conclusion:**

In patients with COVID-19, CD contributed to a worse clinical course, but it did not induce significant changes in LUS. Our findings suggest that pathophysiological factors other than those reflected by LUS may be responsible for the differences in clinical condition between CD+ and CD− patients.

## Introduction

The coronavirus disease 2019 (COVID-19) may present as severe acute respiratory syndrome [1, 2]. Approximately 17–35% of the hospitalized patients are admitted to the intensive care unit (ICU) due to respiratory failure, often receiving invasive mechanical ventilatory support (IMV) [1, 3].

Radiologic features show parenchyma destruction and progressive extension during COVID-19 pneumonia [4, 5]. Post-mortem examination of the lungs of these patients showed severe endothelial injury, vascular thrombosis and microangiopathy of alveolar capillaries with diffuse alveolar destruction [6]. Bedside lung ultrasound (LUS) has been validated as a diagnostic and monitoring tool [4, 7]. LUS allows identifying diffuse alveolar–interstitial syndrome with pleural and pulmonary parenchyma impairment signs, which may be gathered into a score, helping categorize pneumonia severity [7, 8]. LUS scores have been shown to predict the duration of mechanical ventilation [9], treatment response [8] and prognosis [10].

COVID-19 pneumonia has a negative impact on cardiac function, and COVID-19 patients with the burden of cardiac dysfunction (CD) have high mortality and morbidity rates during pneumonia progression [11, 12]. Also, COVID-19 non-survivors with echocardiographic signs of CD had higher LUS scores than COVID-19 disease survivors [13]. It is known that some LUS findings, such as B-lines, characteristic of interstitial pneumonia, may be worsened by cardiogenic alveolar edema [14, 15]. Therefore, we hypothesized that LUS abnormalities might correlate with the severity of the COVID-19 patients. We considered that characterizing the LUS findings in COVID-19 patients with CD (CD+) and those without CD (CD−) would help improve our understanding of how CD modifies the clinical course of SARS-COV 2 infection. We hypothesized that, in patients with low cardiac reserve [16], a higher number and sum score of LUS abnormalities would be found at the time of COVID-19 infection, to decrease with pneumonia resolution. Initially, CD+ patients may show LUS findings compatible with cardiogenic alveolar edema (i.e., confluent B-lines), confounding the prognosis value of LUS findings (score) in COVID-19 pneumonia.

## Material and methods

Adult patients with acute respiratory failure were recruited from June 1 to September 15 of 2020. COVID-19 pneumonia was defined as: (1) positive test for SARS-CoV-2 ribonucleic acid by real-time polymerase chain reaction, collected in nasopharyngeal swab specimens; and (2) evidence of lower respiratory disease by clinical assessment and chest imaging compatible with COVID-19 respiratory failure. The Ethics Committee of the Hospital Garcia de Orta E.P.E approved the study protocol (number 30/2020). Subjects signed a digital informed consent before inclusion in the study. In case of cognitive impairment, the Ethical Committee was contacted, and the necessity for informed consent was waived.

Data were prospectively collected, including demographic data, past medical history, clinical symptoms and signs, laboratory findings, and treatment. Past medical history was collected, focusing on previously established cardiac disease defined by the presence of coronary artery disease, heart failure, cardiomyopathy, and arrhythmias. The following tests were systematically repeated three times along the course of the patient’s hospital stay: first day (D1), fifth day (D5) and tenth day (D10). All patient data were anonymized to ensure blind off-line analysis.

### Laboratory and chest X-ray

Laboratory assessments consisted of a complete blood count, coagulation testing, liver and renal function assessment, electrolytes, and acute inflammatory enzymes such as C-reactive protein, procalcitonin, and ferritin. We also obtained the ratio of the arterial partial pressure of oxygen to the fraction of the inspired oxygen (P/F ratio).

Chest X-rays were reviewed by a senior radiologist (author AG) and classified according to the British Society of Thoracic Imaging [17] recently validated [18] for the COVID-19 disease categorization (Classic COVID-19 or Not Classic of COVID-19).

### Echocardiography

As described elsewhere, problem-oriented focused echocardiography (FoCUS) was performed using an emergency setting protocol [23]. A sectorial probe was used (S4-1 MHz, Philips Lumify, USA), and electrocardiographic monitoring was omitted. A clip of 5-s duration was recorded using parasternal short and long-axis view, apical four-chamber view and subcostal view with the patients lying in a supine position. We evaluated the following items: (a) left ventricular (LV) systolic dysfunction, classified as present if the examiner visually estimated a decrease in the ejection fraction; (b) right ventricular (RV) dilatation, classified as present if the size ratio RV/LV was above 1; (c) presence of pericardial effusion; and (d) the observation of inferior vena cava variability with the respiratory cycle (by M-mode), classified as abnormal if collapsed with size variability above 50% during inspiration, or enlarged above 2.1 cm diameter with size variability beneath 50%. These data were used to define two groups of patients: CD+ when either LV systolic dysfunction and RV dilatation were present and CD− when none of these abnormalities was observed. A sub-analysis was made for patients who needed IMV.

### Lung ultrasound

**Fig. 1:**
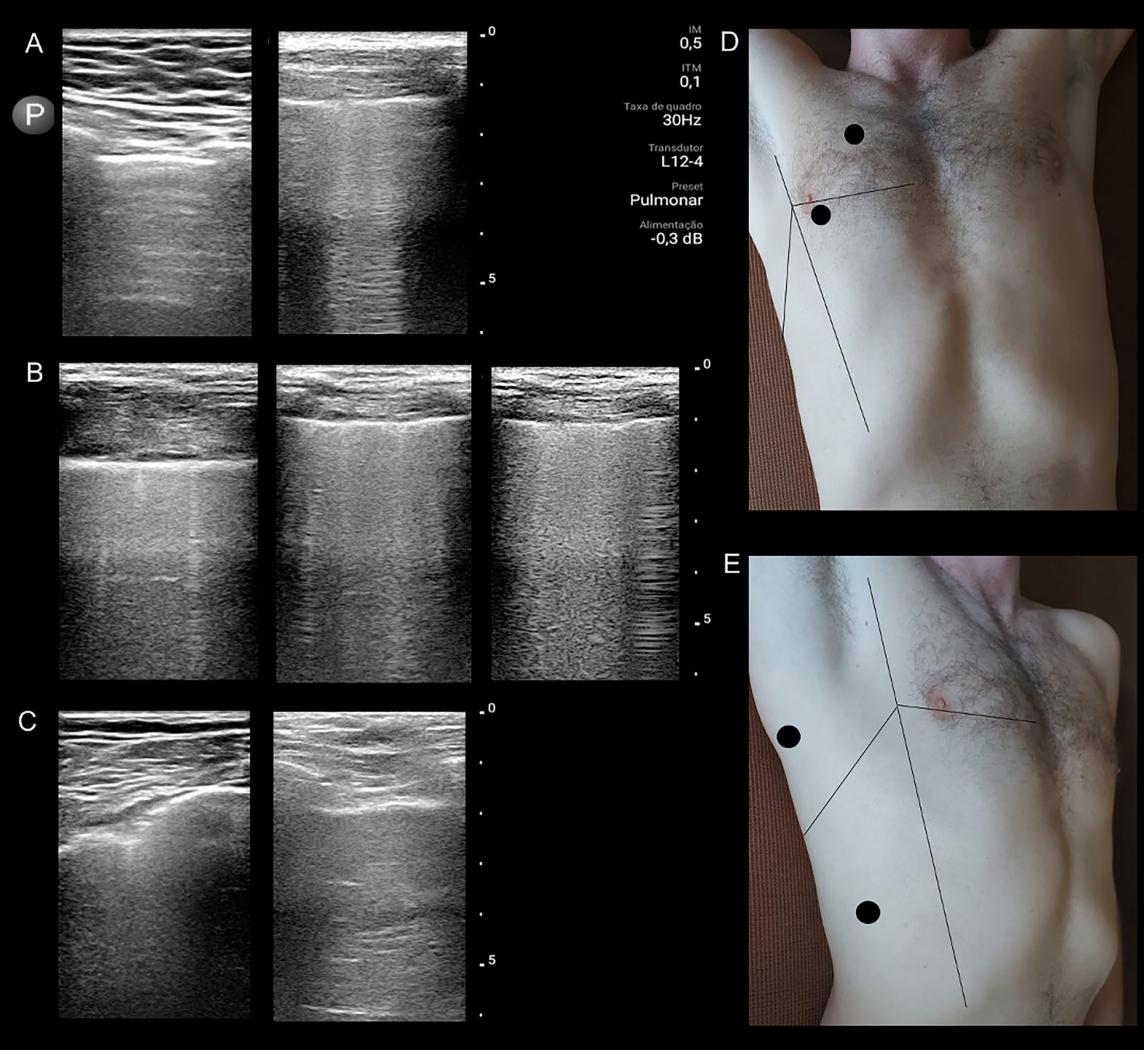
An example of lung ultrasound (LUS) findings and the procedure for calculation of LUS score. Pleura (**A**) was scored 1 point (left image) or 2 points (right). The B-lines presence (**B**) scored 1 points (left), 2 points (middle) or 3 points (right), whereas consolidations presence scored 4 points (left) or 5 points (right). The hemithorax regions evaluated are also shown including the initial probe points at each region (**D** and **E**)

LUS examination was performed using Philips Lumify ultrasound system (linear probe, L12 4 MHz, Philips, USA). Patients were in a semi-sitting position or supine (in the case of IMV). Recordings were made with longitudinal and transversal scans after screening four regions in each hemithorax [19] starting from the points described in the modified bedside lung ultrasound in emergency protocol [20, 21]. Depth was adjusted to 5 cm below the pleural line. We screened two anterior (superior point and M-point) and two lateral regions (posterolateral alveolar and/or pleural syndrome point and diaphragm point) [20–22] and recorded a video clip of 5-s duration for each region. In each video, the following point scoring for abnormalities was made: (a) for pleura, 1 point when it was found thickened (diffuse border) and/or irregular (small echogenic bands); (b) for B-lines, 1 point when one to three regular B-lines were present; 2 points when four to seven irregular B-lines were present, and 3 points when at least four confluent B-lines were present; (c) for consolidations, 4 points when they were present at the subpleural level and 5 points when they were present in the rest of the parenchyma [9, 10]. A simple sum score of abnormalities on the LUS images was calculated, ranging from 0 to 16 points per each of the eight regions examined, up to a possible total of 128 points. A graphic plot of representative abnormal findings is shown in Fig. 1 to facilitate understanding of our methods.

### Data reduction and statistical analysis

Clinical and laboratory parameters were evaluated as continuous variables. Imaging data were described as categorical variables, summarized as counts and percentages, or continuous variables (i.e., LUS score). Descriptive statistics were expressed according to the variable normality distribution (i.e., medians and interquartile ranges if Gaussian distribution was not present). Normality of data was tested with the Shapiro–Wilk test and homogeneity by the Levene test.

The continuous variables were compared using an independent *t*-test, and the dichotomous variables were compared using Pearson’s Chi-square test when appropriate. Correlations for dichotomous and categorical variables were tested with generalized linear models of Spearman correlation and linear regression for continuous variables. Before performing statistical tests, we ensured that the extracted data complied with all assumptions required. Statistical analyses were performed using SPSS Statistics (version 24, IBM, USA). A *p*-value of < 0.05 was considered statistically significant.

## Results

Fifty-one out of the 55 initially recruited patients were included in the study (28 women, median age 62 years ± 17). Exclusions were due to past medical history of pulmonary fibrosis (one patient), technical error importing imaging data set (one patient) and equivocal SARS-COVID tests (two patients). Cohort characteristics are shown in Table 1. At the time of hospitalization, patients had a 5-day median symptom onset, typically fever and their most prevalent comorbidity was hypertension (Table 1). In five patients, chronic obstructive pulmonary disease and stage four chronic kidney disease were present. Of 30 patients admitted to the ICU, eight needed IMV, and sixteen received high-flow nasal cannula. Vasopressors (intravenous norepinephrine) were used in eight patients and kidney replacement therapy in two patients. Most patients received methylprednisolone 1 mg/kg for 10 days, whereas ten received remdesivir (200 mg on the first day, followed by 100 mg/day for 5 days). Laboratory investigations on admission were within normal limits, except for a mild-to-moderate elevation of acute inflammatory enzymes, such as in C-reactive protein (median of 9 mg/dL), ferritin (median of 925 ng/mL) and erythrocyte sedimentation velocity (median of 111 mm per hour). Due to clinical improvement, 21 patients out of the 31 admitted to the ICU stepped down to the Pulmonology department within the study days. The median hospital stay was 14 days. Nine patients died within the first 30 days after hospital admission, most of them (eight patients) between D5 and D10.

**Table 1:** Demographic, clinical and laboratory characteristics of the patients’ cohort

Parameter	CD+	CD−	*p* value
Gender F/M (*n*)	16/6 (22)	12/17 (29)	0.02
Age, median (IQR)	69 (54–79)	59 (49–70)	0.03
Obesity, *n* (%)	10 (46)	10 (35)	> 0.1
Hypertension, *n* (%)	12 (55)	21 (72)	> 0.1
Diabetes mellitus, *n* (%)	5 (23)	13 (44)	> 0.1
Ischemic cardiac disease, *n* (%)	1 (5)	5 (17)	> 0.1
Days since onset of symptoms, median (IQR)	5 (3–6)	5 (3–8)	> 0.1
Lymphocytes (cells/L), median (IQR)	755 (595–1200)	980 (685–1680)	0.04
Neutrophils/lymphocytes ratio, median (IQR)	8 (7–10)	6 (3–8)	> 0.1
CRP (mg/dl), median (IQR)	12 (8–18)	8 (4–13)	0.06
ESR (mm per hour), median (IQR)	120 (74–120)	109 (82–120)	> 0.1
Ferritin (ng/mL), median (IQR)	905 (670–1275)	791 (490–1370)	> 0.1
Troponin (ng/mL), median (IQR)	13(13–23)	13(13–26)	> 0.1
NT-proBNP (pg/mL), median (IQR)	205(69–686)	275(89–980)	> 0.1
Admitted in ICU, *n* (%)	19 (86)	11 (38)	0.004
Invasive mechanical ventilation, *n* (%), median in days (IQR)	7 (32), 6 (5–17)	1 (3), 16	> 0.1
Treatment, *n* (%) Methylprednisolone	19 (86)	20 (69)	0.03
ICU length of stay in days, median (IQR)	12 (8–18)	11 (8–22)	> 0.1
Hospital length of stay in days, median (IQR)	12 (10–20)	17 (8–28)	> 0.1
Mortality, *n* (%) 30 days mortality, *n* (%)	1 (5) 1 (5)	7 (24) 8 (28)	> 0.1 > 0.1

*CRP* C-reactive protein, *ESR* erythrocyte sedimentation rate, *ICU* Intensive care unit, *IQR* interquartile range, *NT-proBNP* N-terminal prohormone of brain natriuretic peptide

### Cardiac dysfunction

From the initial echocardiography examination at D1, 22 patients were included in the CD+ group (43.1%) and 29 in the CD− group (56.9%). Due to the demographic changes reported above, the number of patients included in CD+ and CD− groups differed at each time evaluation point (Table 2).

**Table 2:** Clinical and chest imaging during hospital evaluation time points

Parameter/time	D1			D5			D10		
	CD +	CD−	p value	CD +	CD−	*p* value	CD +	CD−	*p* value
Patients, n	22	29	> 0.1	21	26	> 0.1	16	14	> 0.1
P/F ratio, mean	201 (162–239	258 (222–294)	0.02	189 (152–224)	275 (232–318)	0.003	204 (160–247)	276 (205–348)	0.01
LUS score, mean	31 (26–39)	26 (22–31)	> 0.1	30 (24–37)	27 (23–32)	> 0.1	27 (19–35)	25 (19–31)	> 0.1
Chest X-ray, *n*									
COVID-19	10	14	> 0.1	15	18	> 0.1	11	6	> 0.1
Not classic COVID-19	12	15	> 0.1	6	8	> 0.1	5	8	> 0.1

Data are shown as the number of patients (*n*) or mean and 95% confidence interval (between parentheses)

*CD+* cardiac dysfunction, *LUS* lung ultrasound, *CD−* without cardiac dysfunction

At D1, all 22 patients of the CD+ group showed LV dysfunction. In ten of them (45%), dilated inferior vena cava was also present, and in six (24%), there was additionally RV dysfunction (i.e., biventricular dysfunction). Four of these six patients required IMV. A correlation was found between dilated inferior vena cava and the P/F ratio (*r*_s_ = 0.537; *p* = 0.001). Biventricular dysfunction was found in seven patients at D5 and two at D10.

Mean age was barely significantly higher for CD+ than for CD− patients (*p* = 0.03). The past medical history of cardiovascular disease was similar in both groups. Lymphopenia was significantly more pronounced in CD+ than in CD− patients. A high interquartile range was obtained in both groups in N-terminal prohormone of brain natriuretic peptide, but no between-group differences were found in cardiac enzymes. ICU admission was more prevalent, and IMV was more frequently used in the CD+ than in the CD− group of patients. The percentage of CD+ patients receiving corticoid treatment was higher than that of CD− patients (86% vs 69%, *p* = 0.03). Death occurred more frequently in CD− than in CD+ patients during the hospital stay (*p* > 0.1).

Patients’ mean P/F ratio and chest imaging characteristics are shown in Table 2. A significantly lower mean P/F ratio was found in CD+ than in CD− patients at D1, D5 and D10 evaluation times. Patients of the CD+ group under IMV showed a similar mean P/F to the CD− non-ventilated patients during hospital stay (D1 mean P/F of 211 mmHg, 95% CI 135–287 mmHg). Survivors and non-survivors showed a similar P/F ratio.

### Chest X-ray

Data on chest X-ray examination are summarized in Table 2. On D1, chest X-rays suggested classic COVID-19 pneumonia in 24 patients (47%), increasing through D5 for 33 patients (65%). No significant differences were noted between groups. In addition, classic COVID-19 chest X-ray did not correlate with P/F ratio or LUS score after controlling for age, CD, and IMV.

### Lung ultrasound findings

Data extracted from LUS examinations are reported in Table 3 for a total of 2340 abnormalities noted in 141 scans. Pleural thickening was the most prevalent finding (43%), followed by the presence of irregular and confluent B-lines (24%). Lobar and subpleural consolidations (13%) were more prevalent in all patients' lower regions of the thorax. In non-survivors, pleural irregularities were the most prevalent finding (54%), followed by B-lines (33%). Subpleural and lobar consolidations were 10% in non-survivors (nine patients).

**Table 3:** Number of LUS abnormal findings during the hospital stay and score sum per region

LUS findings	Right hemithorax						Left hemithorax					
	Upper part			Lower part			Upper part			Lower part		
	CD+	CD-	p value	CD+	CD-	*p* value	CD+	CD-	*p* value	CD+	CD-	*p* value
Pleura												
Irregular	117	135	<0.01	119	142	<0.01	109	136	<0.01	118	144	<0.01
B-lines												
Regular ≤ 3	57	74	>0.1	51	69	>0.1	42	50	>0.1	38	53	>0.1
Irregular ≤ 7	49	57	>0.1	44	56	>0.1	60	62	>0.1	59	50	>0.1
Confluent ≥ 4	7	7	>0.1	21	10	>0.05	14	10	>0.1	20	18	>0.1
Consolidations												
Subpleural	19	40	0.01	40	54	>0.1	40	32	>0.1	42	36	>0.1
Lobar	3	2	>0.1	5	5	>0.1	5	4	>0.1	10	9	>0.1
Total, number (%)	247 (24)	315 (25)	0.04	280 (25)	337 (27)	>0.05	270 (25)	294 (23)	>0.1	287 (26)	310 (25)	>0.1

Each hemithorax was divided into upper and lower parts, including LUS findings of one anterior and one lateral region per part. According to their cardiac dysfunction (CD+ and CD−), findings were presented after patients division in two groups

**Fig. 2:**
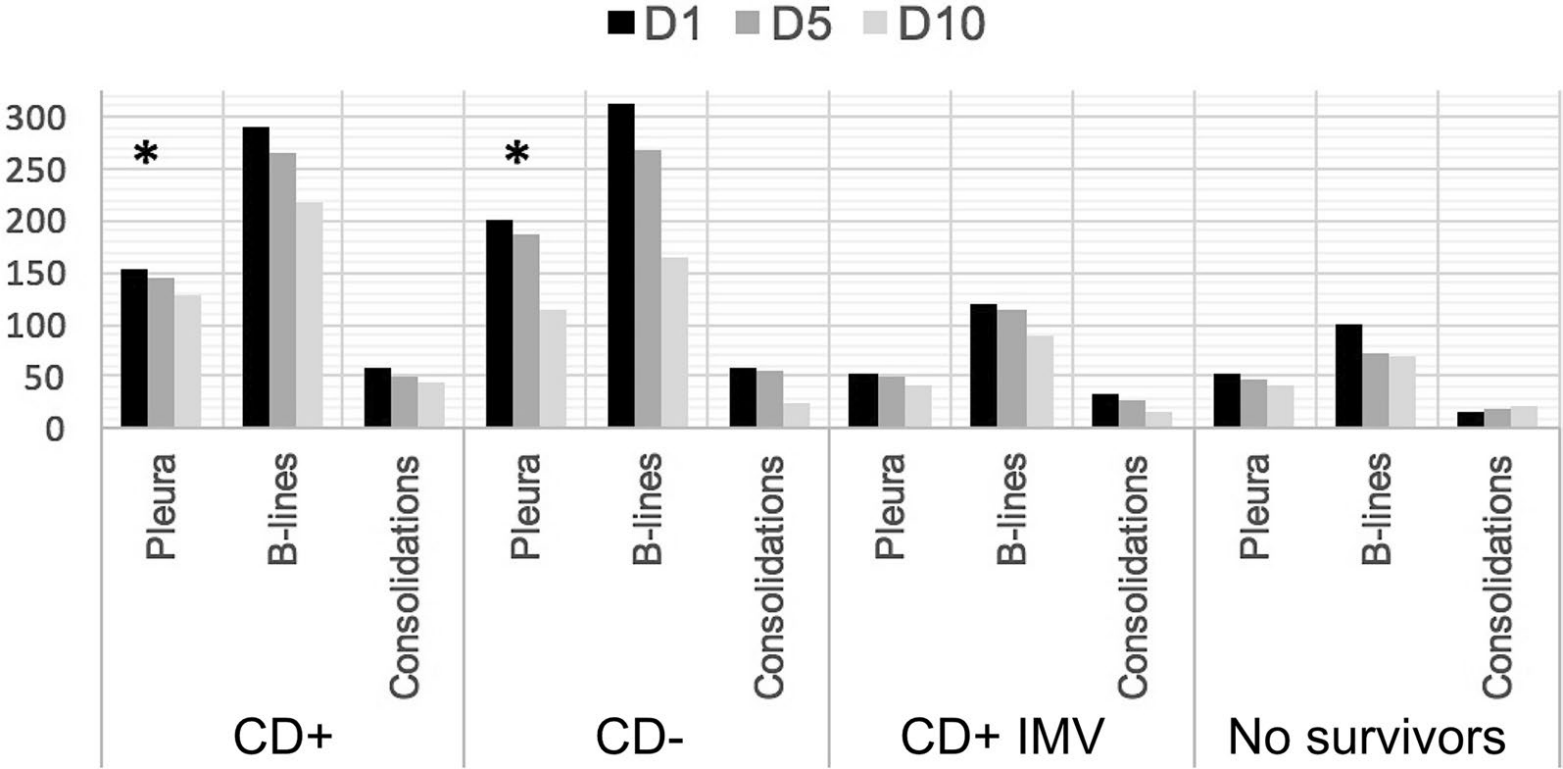
Number of abnormal LUS findings during hospital stay evaluation time points D1–D10 in all groups of patients: CD+  = patients with signs of cardiac dysfunction; CD−: patients with no signs of cardiac dysfunction; CD+ IMV = CD+ patients who required IMV; no-survivors = patients who died during their stay. All types of B-lines evaluated were grouped. The number of patients included is different for each time point (Table 2). *Statistical difference between groups (*p* < 0.05)

There were statistically significant differences between groups in the number of pleural irregularities, significantly higher in all examined sites in CD− than in CD+ patients. There were also more consolidation signs and the total number of LUS signs found in the upper part of the right hemithorax in CD− than in CD+ patients. The total number of subpleural consolidations was significantly higher in CD+ patients requiring IMV than in those not requiring IMV (73 vs 53; *p* = 0.03), and the prevalence of pleural irregularities in CD+ patients was significantly higher in those not requiring IMV than in those requiring IMV (287 vs 169; *p* = 0.0001). A decrease in LUS abnormalities was observed in both groups through the study time points (Fig. 2).

Data on LUS scores are summarized in Table 2. The mean score after pooling together data from the two groups at D1 was 29 (95% CI between 25 and 33). Patients admitted to ICU showed a mean LUS score of 34 (95% CI 29–38) compared to 22 (95% CI 16–28; *p* = 0.01) observed in patients not admitted to the ICU. At D10, LUS score positively correlated with patients' hospital stay length (*r*_p_ (48) = 0.34, *p* = 0.016) after controlling for age and gender. There were no differences in LUS scores between CD+ and CD− patients across time points, despite a lower number of pleural irregularities and subpleural consolidations in the upper part of the right hemithorax in CD+ than in CD− (Table 2).

**Fig. 3:**
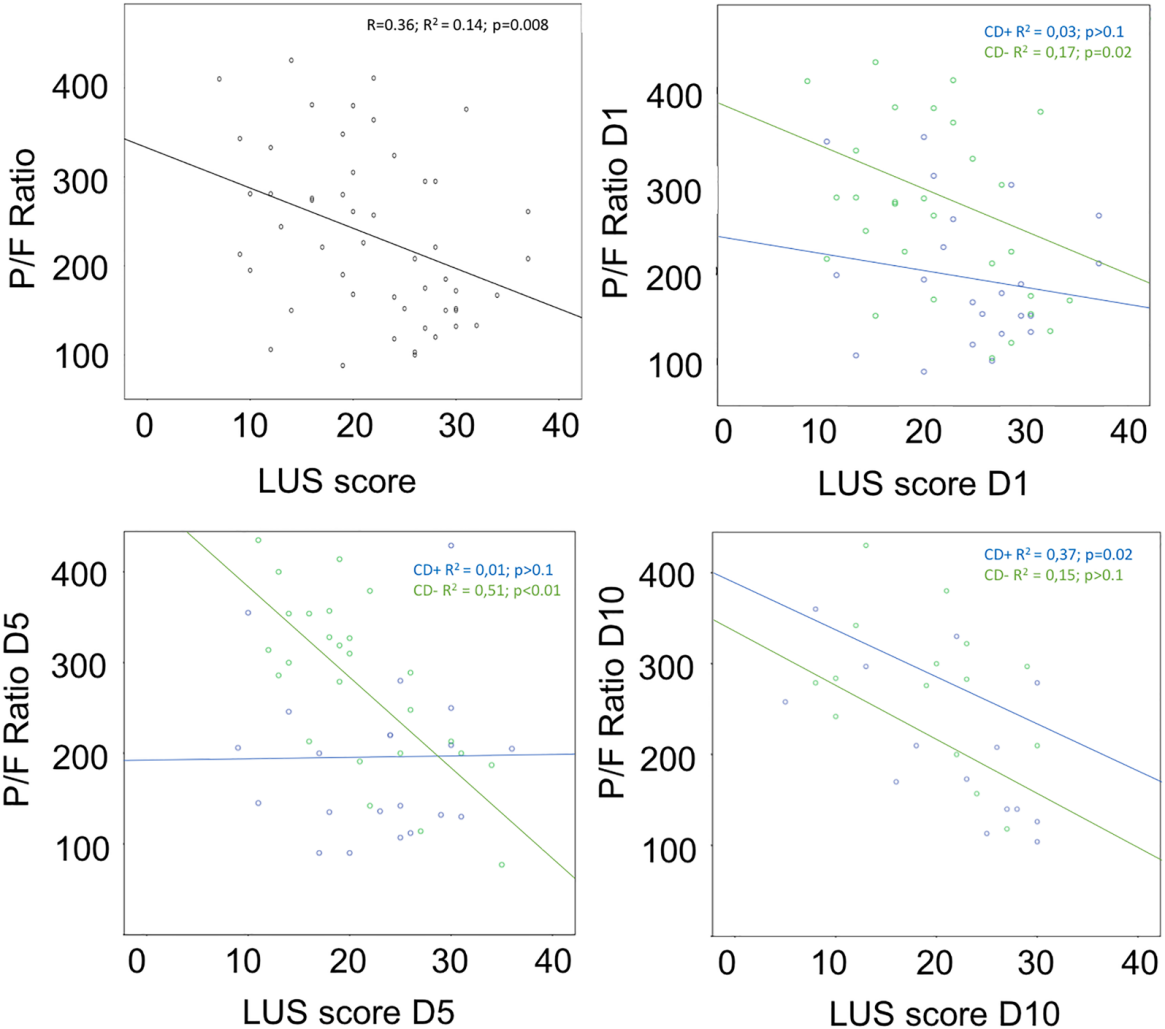
Correlation between lung ultrasound (LUS) score and the arterial oxygen partial pressure times fractional inspired oxygen ratio (P/F ratio) at D1, D5 and D10 evaluation times. Linear relation showed in overall cohort (top left image) and after dividing patients according to the presence of cardiac dysfunction (CD + and CD−). The number of patients included in each evaluation is shown in Table 2. Note that LUS of CD + group only correlated with LUS findings at D10 evaluation

The results of correlation analyses between cohort LUS scores and P/F ratios at admission and across the study times are shown in Fig. 3. Overall, a statistically significant correlation was found in all patients pooled together (*r* = 0.36; F[1,49] = 7,64; *p* = 0.008). LUS score accounted for 14% of the P/F ratio variation (adjusted R [2] of 12%), and a coefficient of 2.2 (95% CI between −7.8 and −1.2) was predicted to obtain the following regression line equation:

1 $$ \frac{P}{F}{\kern 1pt} \,{\text{mmHg}} = 333\, {\text{mmHg}} - \left( {4.7 \times {\text{LUS score}}} \right) $$

A subgroup analysis showed a statistically significant negative correlation between LUS score and the P/F ratio in CD− patients from admission until D10, accounting for 51% of the P/F ratio in D5. In contrast, CD+ patients only showed a significant correlation in D10.

Patients of the CD+ group requiring IMV had higher LUS scores than CD+ patients not requiring IMV, from D1 (mean of 46, 95% CI 35–57 vs 26, 95% CI 22–30, *p* = 0.001) to D5 (mean of 40, 95% CI 34–47 vs 25, 95% CI 22–33, p = 0.01). LUS scores obtained in D10 and D15 were similar in both groups.

Non-survivors and survivors showed similar mean LUS scores at admission and along with the time points when tests were possible.

## Discussion

Our study characterized longitudinally the influence of CD, identified by echocardiography (LV or RV dysfunction), on LUS findings during moderate-to-severe COVID-19 pneumonia. CD+ patients presented fewer LUS pathologic findings than CD− patients in the upper part of the right hemithorax, but the LUS scores were not different between groups. Furthermore, there was a better correlation between LUS scores and P/F ratio in CD− than in CD+ patients during the hospital stay. However, the clinical condition of CD+ patients was more severe, as they had a worse P/F ratio, more ICU admission, and required IMV more frequently than CD− patients. [26]

### Cardiac dysfunction

SARS-CoV 2 infection promotes a systemic inflammatory response [11]. In an advanced stage, a cytokine storm may progress with myocardial injury, manifested by heart failure and symptomatic CD. CD has been already reported to occur after acute SARS-COV 2 infection [24]. In our study, CD detected with FoCUS was present without previous cardiovascular disease despite a high prevalence of cardiovascular risk factors in COVID-19 patients, as has already been reported [13, 24, 25]. In our study, only coronary artery disease, heart failure, cardiomyopathy, and arrhythmias were considered cardiovascular disease (i.e., six patients).

About half of the patients in this study showed CD, with LV and biventricular dysfunction in 43% and 24%, respectively. A similar percentage of CD patients with COVID-19 pneumonia was found by Lazzeri and colleagues (2021) [13] evaluated by increased RV/LV ratio in non-survivors than in survivors. A comprehensive echocardiographic study also found a similar frequency of CD in COVID-19 pneumonia [28]. Furthermore, Li and colleagues (2021) [31] found RV dysfunction in 27 out of 89 patients with cardiovascular disease and without elevation of cardiac biomarkers. In addition, a significant increase in cardiac biomarkers has been reported in only 30% of hospitalized COVID-19 patients [29] with established cardiovascular disease [29–31].

Pulmonary vasoconstriction with thrombotic vessel changes occurs during SARS-CoV 2 infection contributing to increased pulmonary systolic arterial pressure [6, 12, 27], although it may not occur due to low afterload stress in non-ventilated patients. [27] Another potential explanation is that patients hospitalized with COVID-19 had coronary heart disease before infection with SARS-CoV-2. [31]. However, in one series, the total percentage of patients with known coronary heart disease was 10.6%, and only 29.3% of those with elevated troponins had a history of known coronary heart disease [30]. Therefore, other potential mechanisms are likely to have a role in cardiac injury [30].

In our study, both groups showed a high interquartile range of the N-terminal prohormone of brain natriuretic peptide, surpassing the lower cutoff to exclude CD in patients with acute dyspnea [34]. Taking into account such data, we could hypothesize that an unbalanced pro-inflammatory milieu, rather than a direct cytopathic effect on myocytes, would lead to a cardio-depressor effect as in sepsis [34]. However, only half of our cohort showed CD. We hypothesized that some patients might develop a myocarditis-like injury, resulting from either direct infection of the cardiac myocytes or infection of non-myocytes [32]. In fact, an association was recently described [32] between the immunological trait and CD in severe COVID-19 pneumonia [33]. This could also explain the increase in cardiac function across time points simultaneously with the solving COVID-19 infection. [35]

### Chest imaging

Chest X-ray is a first-choice imaging modality for evaluating patients with respiratory distress, but lacks sensitivity for diagnosing COVID-19 pneumonia [35–38]. In our study, we used the British Society of Thoracic Imaging guidelines for categorizing chest radiographs [17]. Our results showed a similar sensitivity to identify classic COVID-19 pneumonia features in about half of the patients. Literature reports support our data [36, 37]. Classic COVID-19 chest X-rays findings increased throughout the hospital stay, which could be explained by the time needed for neutrophil and macrophage alveolar infiltration establishing a radiological opacity [38].

LUS is a useful first tool in an emergency setting to help diagnose COVID-19 pneumonia, distinguishing mild from severe forms of acute respiratory distress syndrome [10, 39–41], and monitoring disease progression [7, 9, 12, 42–46]. The presence of bilateral B-lines, white lung areas with patchy peripheral distribution and sparing areas is the most suggestive LUS picture of COVID-19 [41]. Furthermore, LUS showed good agreement between confluent B-lines and subpleural consolidations findings with computed tomography in identifying ground-glass opacity and peripheral consolidations on lung parenchyma, respectively[4, 5, 39–45]. Moreover, such knowledge is based on a daily increase of LUS by intensivists compared with the pre-COVID-19 era [47].

In our patients, the most common finding was bilateral pleural thickening followed by B-lines and consolidations in the lower part of the thorax. Patients in the CD+ group showed fewer pleural irregularities and subpleural consolidations if not requiring IMV, with an overall sparing of the upper part of the right hemithorax than patients in the CD− group. Similar COVID-19 pneumonia LUS findings have been already reported [13, 33], but none described LUS specifically concerning CD, nor their evolution with treatment during the hospital stay. In our cohort, LUS findings prevalence improved along with successive time points, predominantly in CD− patients, consistent with other patients’ series [13, 46]. In contrast, non-survivors showed an increase in the number of consolidations at D10.

Cardiogenic pulmonary edema can present in LUS as regular homogeneous B-lines in continuous regions of the lung parenchyma [14, 15]. These findings are differentiated from the typical pattern of progressive bilateral loss of alveolar aeration in moderate-to-severe COVID-19 pneumonia, starting from isolated B-lines to confluent B-lines and consolidation findings [20, 22, 39, 41]. In our data, CD+ patients were associated with worse clinical condition than CD− patients, as they required IMV more frequently and had significantly lower P/F ratios. However, despite the high prevalence of LV dysfunction in our sample, the presence of B-lines was not higher in CD+ than CD− patients. Such findings may suggest a predominance of pneumonic alveolar edema. Ultrasound cardiogenic alveolar edema is reflected by the presence of homogenous B-lines in lung regions with low ventilation–perfusion ratio [15] (i.e., lower lung regions), sparing the upper thorax regions, as shown by our data.

LUS score in COVID-19 pneumonia patients correlated with prognosis [7, 9, 10, 12, 46]. LUS score is calculated to demonstrate lung involvement and alveolar destruction [9, 10, 46, 48]. Some B-line signs may be related only to COVID-19 pneumonia [39]. In our study, we based our score on the progressive loss of aeration described in pneumonia [20, 39, 48]. However, we discriminated between subpleural and lobar consolidations as the most relevant finding [10, 38, 43] due to their contribution to uneven alveolar ventilation–perfusion [8, 25, 27]. Our data showed that LUS scores obtained at D1 predicted the P/F ratio when using comprehensive cohort data. As reported in the literature, such findings were more pronounced in CD− patients during their hospital stay [42, 46]. In CD+ patients, we found no correlation between LUS scores and the P/F ratio until D10. Such results and the fewer LUS findings and their distribution in CD+ patients may be explained by the predominant presence of cardiogenic alveolar edema. On the other hand, CD+ patients requiring IMV had significantly higher LUS scores than CD+ patients not requiring IMV, which the predominant presence of pneumonia-related findings may explain.

## Limitations

Some limitations may have an impact on our study results. We had older and predominantly male patients in the CD+ group. Because our study was prospective and not selective, the difference may suggest that older males are more prone than young females to present CD, independently of having COVID-19. Regarding severity, only moderate-to-severe forms of COVID-19 pneumonia were included in our study, which may invalidate outcome prediction in other severity ranges [5]. In part, due to these limitations, both type I and type II errors may occur, influencing the score applicability. A larger sample of patients would be desirable for accurate clinical outcome prediction [8]. However, even with these limitations, we could show that the LUS score predicted the P/F ratio in CD− patients.

We assessed CD on the bases of FoCUS, without grading the dysfunction [12, 24]. This limitation and the absence of significantly elevated cardiac biomarkers may have influenced our findings. FoCUS clips were recorded and reviewed repeatedly to minimize such disadvantages. However, the portable ultrasound devices allowed to maintain contact and droplet precaution during longitudinal evaluation, which helped characterize COVID-19 pneumonia natural history.

## Conclusion

In summary, about half of the patients with moderate COVID-19 pneumonia showed FoCUS signs of CD. Lung parenchyma involvement assessed by LUS showed lower pleural thickening and subpleural consolidations in CD+ patients not requiring IMV than in CD− patients. In fact, despite the high prevalence of LV dysfunction, the presence of B-lines was not higher. However, a lower P/F ratio was observed in CD+ than in CD− patients, contributing to a higher rate of ICU admission and IMV requirement. Overall, two clinical phenotypes of CD+ patients were observed. Those needing IMV (i.e., severe pneumonia), which presented a low P/F ratio and increased LUS scores, and those with moderate pneumonia, with low P/F and low LUS scores, which improved P/F and the correlation between P/F and LUS score after treatment, suggesting the presence of cardiogenic alveolar edema.

## Data Availability

All data and materials are available on request.
